# Controlled vocabularies for plant anatomical parts optimized for use in data analysis tools and for cross-species studies

**DOI:** 10.1186/1746-4811-9-33

**Published:** 2013-08-06

**Authors:** Rasa Meskauskiene, Oliver Laule, Nikolai V Ivanov, Florian Martin, Markus Wyss, Wilhelm Gruissem, Philip Zimmermann

**Affiliations:** 1 Department of Biology, ETH Zurich, Zurich 8092, Switzerland; 2 NEBION AG, Hohlstrasse 515, Zurich 8048, Switzerland; 3 Philip Morris International R&D, Quai Jeanrenaud 5, Neuchatel 2003, Switzerland

**Keywords:** Plant anatomy, Controlled vocabularies, Ontology, Database

## Abstract

**Background:**

It is generally accepted that controlled vocabularies are necessary to systematically integrate data from various sources. During the last decade, several plant ontologies have been developed, some of which are community specific or were developed for a particular purpose. In most cases, the practical application of these ontologies has been limited to systematically storing experimental data. Due to technical constraints, complex data structures and term redundancies, it has been difficult to apply them directly into analysis tools.

**Results:**

Here, we describe a simplified and cross-species compatible set of controlled vocabularies for plant anatomy, focussing mainly on monocotypledonous and dicotyledonous crop and model plants. Their content was designed primarily for their direct use in graphical visualization tools. Specifically, we created annotation vocabularies that can be understood by non-specialists, are minimally redundant, simply structured, have low tree depth, and we tested them practically in the frame of Genevestigator.

**Conclusions:**

The application of the proposed ontologies enabled the aggregation of data from hundreds of experiments to visualize gene expression across tissue types. It also facilitated the comparison of expression across species. The described controlled vocabularies are maintained by a dedicated curation team and are available upon request.

## Background

The wide-spread use of controlled vocabularies to annotate biological experiments holds the promise of full integration and comparability between experiments and databases. It provides the basis for classifying samples more easily into discrete categories and it also facilitates the exchange of information between databases. Ultimately, improved data integration will lead to more powerful tools providing deeper biological insight. Despite these obvious benefits, ontologies have often been perceived as a necessary pain rather than useful instruments. The main drawback has been that ontologies are not easy to use and require some understanding of their semantics. As a result, few databases exist that make systematic use of ontologies, and many biologists still prefer describing their experiments with plain text rather than using controlled vobacularies. In fact, because in many public databases the submission of experiments is carried out by the experimenter and not by a trained curator, the majority of sample annotations is submitted as plain text. The subsequent transformation of this information into systematic annotations is tedious and the use of controlled vocabularies in the first place must be encouraged. This requires the availability of controlled vocabularies that can be easily interpreted and used by experimenters.

Existing ontologies suffer from a number of weaknesses, such as the use of uncommon or abstract terms, excessive tree depth, and term redundancies that cause confusion among users. For example, ‘cardinal organ part’ or ‘collective plant structure’ may be semantically useful terms, but they are not understood by the majority of biological researchers. A further problem is the attempt to incorporate multiple dimensions of an experimental design into a single ontology, such as anatomical parts, stages of organism development, and perturbations. As a result, some of the existing ontologies are very extensive and possess tree depths that can exceed a dozen levels. This depth represents a major handicap for their use in analysis tools alongside a data matrix. The same holds true for term redundancies, which impair a proper clustering of e.g. a data matrix composed of genes and anatomical parts. An example of term redundancy can be found in the current Plant Structure Ontology [1], in which the term ‘endosperm’ can be found at three locations (in ‘sporophyte/infructescence/fruit/seed/’, in ‘sporophyte/seed/’, and in ‘/tissue/’). This representation is impractical in the context of data analysis because a category may occur multiple times in a given data matrix.

In this work, we present a simplified but precise set of controlled vocabularies for plant anatomy that were developed specifically for their use together with data analysis tools. The vocabularies are based on publicly accepted terms and ontologies from the Plant Ontology Consortium (POC) [2,3], Gramene [4], MaizeGDB [5], and The Arabidopsis Information Resource (TAIR) [6], and their classification structure was built to be cross-species compatible. For the purpose of using them directly in an analytical context, the proposed ontologies had to fulfill the following requirements: 

1. Description of a single dimension of expression (e.g. anatomical parts as representing the spatial dimension).

2. Minimization of the depth of trees that classify anatomical parts. This constraint is to allow placing an ontology tree next to a results plot (e.g. heat map or scatterplot) on average-sized computer screens, and to improve legibility.

3. Avoidance of term redundancies to ensure that every expression vector is represented only once in a given data matrix.

4. Similarity of tree structures between species to facilitate cross-species comparisons.

5. Use of community-accepted vocabularies.

## Results and discussion

### Target communities and purpose

The aim of this work is to help software developers and database curators build intuitive data annotation systems and user-friendly analysis tools incorporating plant anatomy annotations. We therefore constructed and made available a set of simplified, application-driven controlled vocabularies. The intent is not to propose an alternative to ontologies provided by the Plant Ontology Database [2], but to make available a set of controlled vocabularies for a specific use case. The level of detail remains at the granularity of sample sizes that are harvested for protein or gene expression profiling. The proposed ontologies were optimized for agronomically relevant crop species and model plants, with a focus on practical applications. It therefore does not aim at giving a general model for plant structure of both flowering and non-flowering plants.

The proposed controlled vocabularies were developed for monocotyledonous (monocot) and dicotyledonous (dicot) species. Each set of controlled vocabularies is structured as a tree, with parent nodes representing structures that contain multiple organs, tissues or cell types. To ensure cross-species comparability, we developed three basic types of trees: a cross-monocotyledonous tree, a cross-dicotyledonous tree, and a generic plant (angiosperm) anatomy tree that provides the overal basic structure for both monocots and dicots.

### Cross-monocotyledonous tree structure

**Figure 1:**
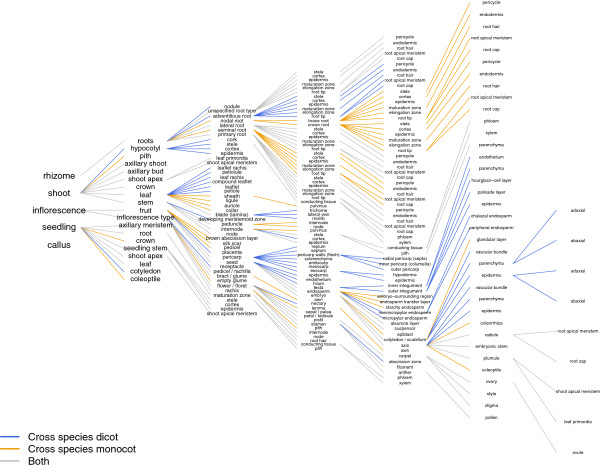
**Generalized trees of anatomical parts for monocotyledonous plants (orange edges), dicotyledonous plants (blue edges), and a general model for monocot-dicot (angiosperms) (gray edges).** The trees were derived from the existing Genevestigator anatomy trees for rice, wheat, barley, maize, Arabidopsis, soybean, tomato, and tobacco.

The generalized tree of anatomical parts for monocots was built using elements from the previously published Genevestigator anatomy trees for rice, wheat, barley, and maize [7]. The hierarchical tree structure, already used in the species-specific anatomy trees, was adapted for the ‘monocot’ anatomy tree. The species-specific and the generalized trees consist of parent and child categories, child anatomical structures being a part of the parent structures in most cases. For example, in the four above mentioned species, spikelet is a part of inflorescence, floret is a part of spikelet, stamen is a part of floret and anther is a part of stamen. Thus, in both anatomy trees (species-specific and generalized), spikelet is a child of inflorescence and the parent of floret; floret is a child of spikelet and the parent of stamen and stamen is a child of floret and the parent of anther (Figure 1). In a few cases, however, the child structure is not a part of a parent structure. For instance, primary, seminal, lateral, and nodal roots are rather root types and not parts of roots (Figure 1). In a few instances, for reasons of experimental setup, some anatomical categories had to be introduced that do not exist in plants, e.g. unspecified root type. These artificial categories are needed for the annotation of samples for which structures, e.g. root tips, of several root types have been combined. Such artificial categories were retained in the ‘monocot’ tree (Figure 1).

In species-specific and the generalized ‘monocot’ trees, it was tried to avoid redundancies such as placing an anatomical structure under more than one parent category. However, identical anatomical structures found in several different organs were placed under the respective parent structures. For example, the mesocotyl, stem internodes, and all root types (primary, lateral, nodal) contain an epidermis. Epidermis is therefore a child category of several parent categories, meaning e.g. ‘epidermis of a primary root’, ‘epidermis of a lateral root’, ‘epidermis of a crown root’, ‘epidermis of a stem internode’, etc. The anatomical structures present in at least one of the four species are basically included in the ‘monocot’ tree. For instance, the ‘monocot’ tree contains pulvinus of stem internode, husk leaf, and brace root although these structures, present in maize, are not found in rice, wheat, or barley (Figure 1). However, the ‘monocot’ tree does not include the male and female inflorescence branches present in the maize-specific anatomy tree. The reason for this is to avoid redundancies such as having e.g. stamen twice in the anatomy tree: once in the male-inflorescence branch and once in the male-female inflorescence branch.

### Cross-dicotyledonous tree structure

The tree of anatomical parts for dicots (Figure 1) was built by identifying common structures between earlier versions of the Genevestigator anatomy trees for Arabidopsis, soybean, tomato, and tobacco. The basic principles are the same as for species-specific and ‘monocot’ anatomy trees: 1. The hierarchical tree reflects a dicot plant with child structures being part of the parent structure except for various root types. 2. Redundancies are avoided with the exception of anatomical structures/tissues/cell-types that are found in several different plant organs. 3. A few anatomical categories that do not exist in plants but are required for experiment-annotation are added. 4. Anatomical structures that are present in at least one of the four species (e.g. nodule, which is only present in soybean) are included, as long as this does not lead to unnecessary redundancies. One exception from this rule is that the dicot tree does not contain the tobacco-specific categories (Primings, Lugs, Cutters, Leaves and Tips), which indicate the position of a leaf on the stalk, and which are used for tobacco experiment annotation (see case study below).

### Extending and merging tree structures

**Figure 2:**
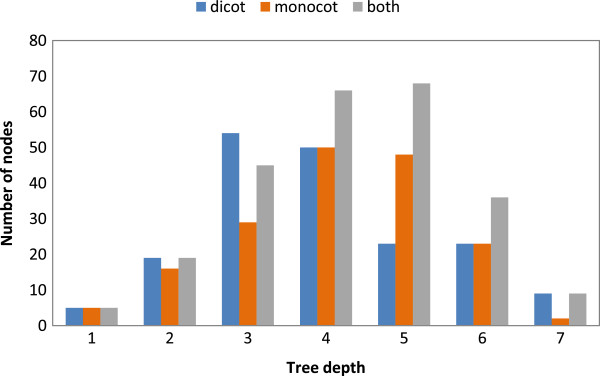
**Distribution of ontology terms as a function of the depth from the root of the tree.** Results are shown for the dicot tree (blue), monocot tree (orange) and the monocot/dicot (angiosperm) tree (grey).

In consideration of two points: 1) which anatomical structures of the generalized tree are not present in the species of interest, and 2) are there anatomical structures present in the species of interest but not in the generalized tree, both the ‘monocot’ and ‘dicot’ anatomical trees can be used to build anatomy trees for further monocotyledonous and dicotyledonous species, respectively. The current ontologies are based on the anatomical structures of a few of species; as new species are added, new categories may appear in the general trees according to the rules defined above. Examples for such extensions and for mappings between monocotyledonous and between dicotyledonous species are provided in Additional file 1 and Additional file 2. Finally, the generic tree of anatomical parts for both dicots and monocots (angiosperms see Figure 2) was built by joining the ‘monocot’ and ‘dicot’ anatomy trees (Figure 1). It contains anatomical structures common to di- and monocots (e.g. lateral root), dicot-specific structures (e.g. seedling cotyledon), monocot-specific structures (e.g. coleoptile), related structures (e.g. sepal / palea; palea, a part of monocot floret, corresponds to petal of dicot flower [8]), and combined structures (e.g. ‘leaf’ meaning various types of leaves, such as flag leaf, rosette leaf, cauline leaf, etc.).

### Applications

With the increasing number of published transcriptome studies and the accumulation of such data in proprietary and public databases, it has become imperative to integrate all experiments using controlled vocabularies. Organizing these vocubularies in trees rather than simple lists of keywords is an important step towards facilitating data interpretation. In fact, the visualization of results for individual tissues in their larger spatial context helps understanding their biological regulation and function.

**Figure 3:**
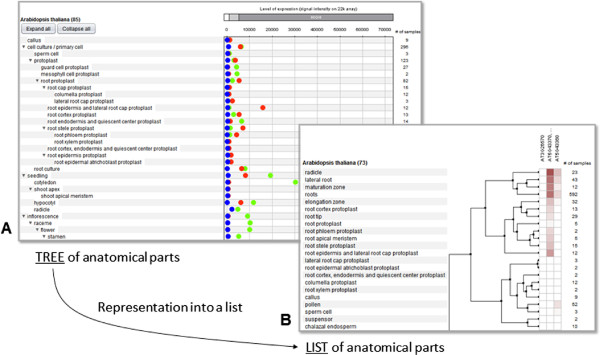
**Example of the implementation of a plant anatomy ontology in a graphical interface.** Expression values from Arabidopsis are plotted against either a tree **(A)** or list **(B)** of anatomical parts. Simple expression values are preferably plotted against a tree which can be expanded/collapsed in a fine-granular way. In this case, the expression value for a given node is the average over all samples within and below that node. In contrast, clustering of the data matrix requires the linearization into a list and the removal of nodes that contain no samples. The above list is derived from the anatomy tree for Arabidopsis, structured according to the generalized tree for dicotyledonous species.

The proposed trees of controlled vocabularies were designed specifically for use in graphical user interfaces. In particular cases of data analysis, however, working with trees is not optimal or feasible. For example, to cluster expression data by genes and by tissue types, it is not possible to order the categories simultaneously according to the anatomy tree and according to the clustering result. Furthermore, for reasons of the semantics of clustering, the aggregated expression information in parent nodes are not desired. Therefore, a list (rather than a tree) of tissue types with corresponding expression data can be generated using the controlled vocabularies originally organized as trees. An example is illustrated in Figure 3 with the Genevestigator Hierarchical Clustering tool.

### Case study

**Figure 4:**
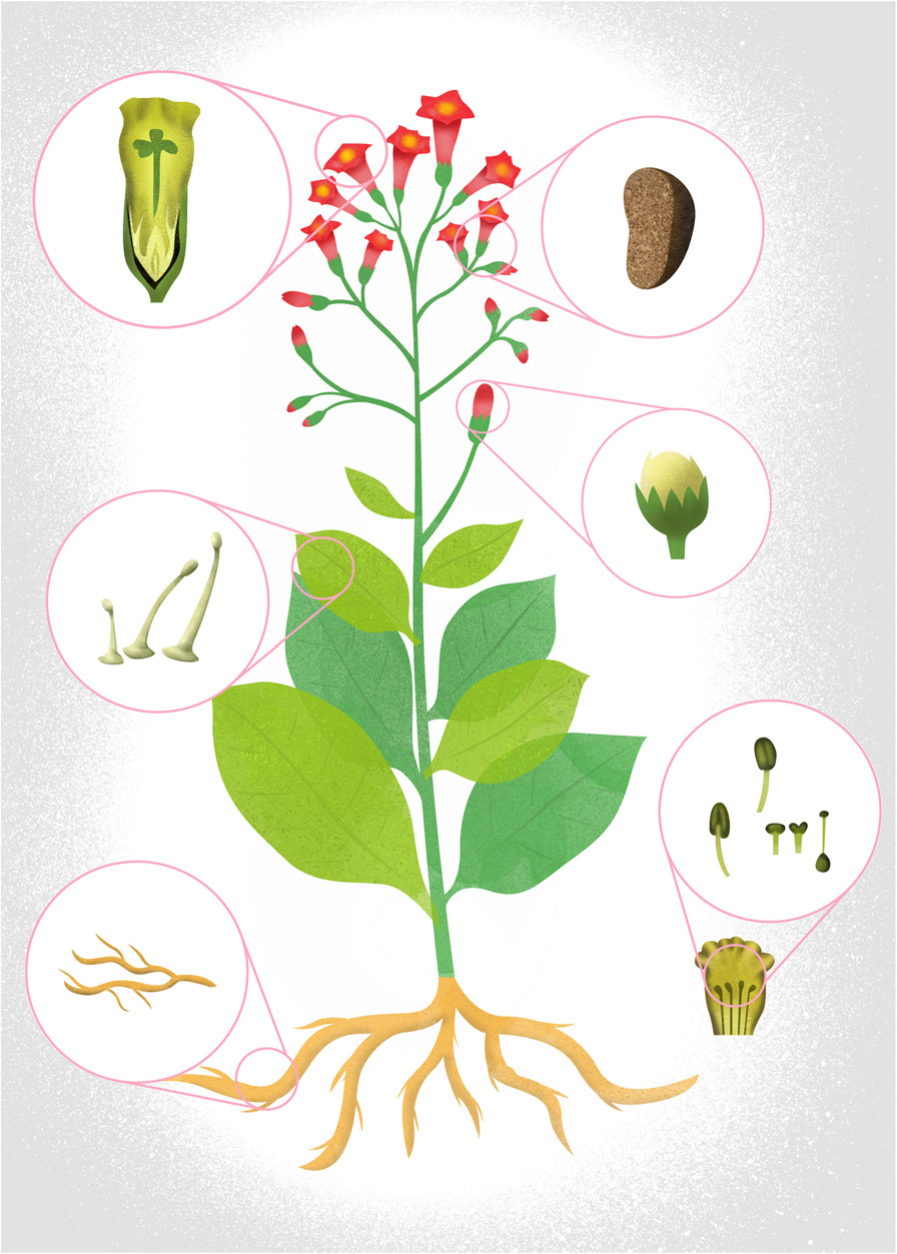
**Anatomical structures of *Nicotiana tabacum*.**

Tobacco (see Figure 4) is both a model plant species and an important industrial crop. The development of an application driven anatomy ontology for tobacco must take consideration of its use. This example illustrates how species specific plant structures were added to the general dicot tree model. In fact, some tobacco specificities were developed to accommodate the plant anatomy with the terminology used in the tobacco industry. The stalk positions have specific names depending on the tobacco type grown (Burley, Oriental or Flue-Cured). This part of the ontology (third level) was developed according to the following terms (from bottom to top): 

1. Burley and Flue-cured: primings, lugs, cutters, leaves, tips

2. Oriental: the stalk anatomy of an Oriental tobacco is quite different, however an equivalence is now widely accepted and the leaf positions are matched to the Burley and Flue-Cured terminology with the exception of primings that do not exist in Oriental varieties.

For each leaf position the ontology includes the subsequent descriptors ‘auxillary bud’ or ‘leaf’ (except for primings where only ‘leaf’ is meaningful), then ‘petiole’ or ‘blade(lamina)’ and finally ‘trichome’ or ‘midrib’ or ‘lateral vein’.

For tobacco, some specificities were included to also describe the curing process, that is part of the tobacco processing after harvest. This aspect was added, as the curing is a key step in studying valued properties of tobacco leaves. It has to be also noted that several microarray experiments ([9]) revealed a gene transcription activity long after ripping. Also greenhouse conditions (e.g lighting, temperature, fertilization, humidity) have been described. The genotype list includes commercial tobacco cultivars from the PMI germplasm collection as well as *N. tabacum* ancestors and other Nicotiana species.

The *N. tabacum* ontology was used within Philip Morris International (PMI) to annotate and describe gene expression experiments for a total of 216 microarrays, as well as for other types of analyses.

Those experiments include: 

1. organ specific (e.g. trichome) studies

2. variety comparison (fields and greenhouse)

3. transcription activity during the curing process (time-course experiment)

4. impact of the cadmium content in soil on the gene signatures

5. cold shock treatment effect on seedlings

6. Nicotiana species comparison (e.g. *N. rustica*)

### Mapping to existing ontologies from POC

To meet community standards, the terms used to describe anatomical structures were mapped to the corresponding POC identifiers. In case of multiple options, the most suitable POC entities were chosen, i.e. our controlled vocabulary terms were mapped to those POC entities where the description applies best. Detailed mappings are available in Additional file 3 and Additional file 4.

In this work, we focused primarily on plant species of agricultural and biotechnological interest. The proposed ontologies were therefore optimized for cereal crops and for dicotyledonous species like Arabidopsis, soybean and tobacco. The choice of using hierarchical trees rather than a more general directed acyclic graph (DAG) was imposed by plot visualization constraints and the need to minimize redundancies. Existing ontologies, such as the Plant Structure Ontology [1] focused primarily on their use to search terms and associated annotations, to identify samples of interest or to associate the expression of particular genes with anatomical parts. Our use case is different, and the adaptations made resulted in ontologies that are slim and purpose-specific, and they work well for the agronomically relevant species described here. As correctly pointed out by Ilic et al. [1], however, for some plant species where a given tissue type can be part of different structures, using a hierarchical system would inevitably result in redundancies. This is rarely the case for the monocotyledonous and dicotyledonous species described here. Therefore, the simplification of a DAG to a hierarchical tree greatly facilitates the implementation of a tree within a tool without causing such undesired redundancies.

The further simplification of the anatomy tree to remove nodes that do not represent physical entities that can be harvested (e.g. terms such as ‘cardinal part’ or ‘collective organ part structure’) resulted in a shallow tree with minimal width. This was essential to facilitate the representation of measurement results in a plot or heat map that is displayed next to the tree. Figure 2 shows the characteristics of the monocot, dicot and general angiosperm tree in terms of tree depth. In contrast to the Plant Structure Ontology [1], which have depths of up to 15 and the most populated depths being 5 and 6, the proposed ontologies have a maximum depth of 8, with the most populated depth being 3 for the dicot model and 4 for the monocot model. Despite this lower depth, the proposed ontologies are sufficiently fine-granular to represent all biological samples that can currently be harvested and genomically profiled. As newer methods of harvesting get closer to single-cell analytics, the granularity will increase while we move from organs to tissues to cell types. The anatomy ontology model described here is extensible and can accomodate new levels. The advent of single-cell profiling is not expected to extend the depth by more than two or three levels.

Currently, the anatomy ontology contains organs and tissues that underwent normal development. It is possible that the same tree structure be used to create a phenotype ontology to capture morphologic variations (quantitative or qualitative). Alternatively, it is conceivable that phenotypic variations get depicted in the same ontology, alongside the corresponding normal anatomical structures to allow direct, side-by-side comparison of gene expression between such structures. Here, we do not impose one or the other way of capturing phenotypic variation into an ontology.

## Conclusions

The ontologies described here have been tested and used practically in the context of a database and analysis tools, namely Genevestigator. The chosen level of structure and granularity has been optimized over several years and provide a robust framework to build user-friendly databases and analysis tools for genomic data. The proposed ontologies are freely available upon request.

## Methods

To build a controlled vocabulary for plant anatomy the following sources were used: Plant Ontology Consortium (POC) [2,3], Gramene [4], MaizeGDB [5], TAIR [6], Food and Agriculture Organization of the United Nations (FAO, www.fao.org), together with other relevant publications [10-12]. For individual plant anatomy trees, terms were chosen according to specificity and acceptance within the respective communities to enable a precise experiment annotation and/or - analysis. For generalized trees (dicots and monocots), however, a more general terminology was applied to facilitate the analysis across different species.

## Competing interests

The authors declare that they have no competing interests.

## Authors’ contributions

OL, WG and PZ designed the project. OL, RM, NI, FM, MW and PZ contributed to developing the ontologies and the software tools for handling ontologies. All authors were involved in writing the manuscript. All authors read and approved the final manuscript.

## Supplementary Material

Additional file 1 Anatomy ontology mapping across various dicotyledonous plant species. Click here for file

Additional file 2 Anatomy ontology mapping across various monocotyledonous plant species. Click here for file

Additional file 3 Mapping of the monocotyledonous plant anatomy ontology to Plant Ontology (PO) identifiers. Click here for file

Additional file 4 Mapping of the dicotyledonous plant anatomy ontology to Plant Ontology (PO) identifiers. Click here for file
